# An Integrated Approach of GRA Coupled with Principal Component Analysis for Multi-Optimization of Shielded Metal Arc Welding (SMAW) Process

**DOI:** 10.3390/ma13163457

**Published:** 2020-08-05

**Authors:** Mohsin Iqbal Qazi, Rehman Akhtar, Muhammad Abas, Qazi Salman Khalid, Abdur Rehman Babar, Catalin Iulian Pruncu

**Affiliations:** 1 Department of Industrial Engineering, Jalozai Campus, University of Engineering and Technology Peshawar, Nowshera 24240, Pakistan; mohsin@uetpeshawar.edu.pk; 2 Department of Industrial Engineering, University of Engineering and Technology Peshawar, Peshawar 25120, Pakistan; rehman_akhtar@uetpeshawar.edu.pk (R.A.); muhammadabas@uetpeshawar.edu.pk (M.A.); qazisalman@uetpeshawar.edu.pk (Q.S.K.); abdurrehman@uetpeshawar.edu.pk (A.R.B.); 3 Department of Mechanical Engineering, Imperial College London, London SW7 2AZ, UK; 4 Department of Mechanical Engineering, School of Engineering, University of Birmingham, Birmingham B15 2TT, UK

**Keywords:** SA 516 Grade 70, SMAW, Taguchi, optimization, grey relational analysis, principal component analysis

## Abstract

Welding distortion is a critical issue as it leads to severe deterioration of structural integrity of welded work piece and dimensional precision. This study aims at studying the effects of shielded metal arc welding (SMAW) parameters on the evolution of mechanical properties, including tensile strength, impact toughness, and hardness, along with angular distortion on a welded joint from SA 516 grade 70. Such parameters are analyzed and optimized by employing the Taguchi method and Grey relational analysis. SA 516 grade 70 is commercially used for fabrication of storage tanks, boilers and pressure vessels. SMAW is investigated with three levels of root gap, groove angle, electrode diameter, and pre-heat temperature, which were varied on a butt joint in flat (1 G) position to determine their effects on response variables at room temperature. Nine experiments were designed using a Taguchi L9 orthogonal array, welded according to American Society of Mechanical Engineers (ASME) section IX, and samples were prepared and tested as per ASTM A 370. The Taguchi method and Grey relational analysis were employed to observe the most significant parameters and optimal levels that synergically yield improved responses. Results are validated by conducting confirmatory experiments that show good agreement with optimum results.

## 1. Introduction

In small to heavy industries, steel is utmost important material for fabrication, structural components, weapons and machines due to low cost, high tensile strength and considerable toughness [1,2]. Shielded metal arc welding (SMAW) is oldest, most rapid, convenient, and commonly used joining process for the fabrication of variety of products such as pressure vessels, gears, machines, ship hulls, mining equipment, boilers, etc. SMAW results in good quality when employed for construction, pressure vessels, military armors, and vehicles [3]. Commercially, due to the low cost and ready availability, SMAW involves a simple setup, versatile source of heat in practice, and is widely used in the welding of steel sections [4]. SMAW is a multi-objective process involving multiple parameters, such as welding speed, electrode diameter, root gap, welding current, groove angle, and polarity, the judicious and precise setting of which results in targeted weld quality [5]. Weld quality is characterized by weld chemistry, as well as the mechanical and metallurgical properties of fusion zone, heat affected zone (HAZ), and bead geometry features. Welding is economical, efficient, and sound when the deposition rate is maximum [6].

Manufacturing of steel structures involves welding as an important phase and fundamental relevance will be assumed in the manufacturing of these technologies. Submerged components, especially in the oil and gas sector impair their proper functionality due to welding discontinuities and defects that are primarily root cause of crack initiation and propagation [7]. Owing to the excellent mechanical properties of SA 516 grade 70 prove it the essential and primary material for the boiler at high working temperatures and good weld-ability. In this regard, these steels got widespread applications in steam generating plants, super heater tubes, and piping. Analytical and experimental design techniques have been widely used for establishing relationships among quality characteristics and process parameters so the desired quality can be fetched efficiently [8]. The microstructure of weld sturdily affects the productivity, integrity, strength, hardness, toughness, and formation of weldment defects.

Ahire et al., applied a genetic algorithm (GA) for optimization of manual metal arc welding (MMAW) process parameters on a dissimilar joint of low carbon steel and stainless-steel SS 304. The experiments were designed by response surface methodology (RSM) to optimize the effect of root gap, welding speed, welding current, and electrode angle on deposition rate and weld strength. They reported that GA significantly improved the process [9]. Ali et al. developed a mathematical model for the SMAW process by employing an artificial neural network, and underlined the effect of preheating, cryo-treatment on weld joint characteristics such as grain growth and refinement, HAZ depth, and weld interface. Authors concluded from literature that heat input directly effects HAZ and penetration which is a function of polarity, travel speed, and current [10]. Bhaduri et al. optimized the tensile strength by investigating the effect of post-weld heat treatment (PWHT) procedures and heat inputs on two microstructures of stainless steel 17-4PH. They concluded that optimum hardness distribution was obtained by using 3.15 mm electrodes that have an intermediate heat input [11]. Osayi et al., attempted to optimize the ultimate tensile strength UTS of the weld joint by employing the Taguchi method fabricated by the MMAW process on low carbon steel AISI 1020. In their investigation welding current was found to be the most significant factor followed by welding speed and root gap respectively [12]. Mirza et al. developed mathematical models and optimized the weld joint properties of various materials such as high strength low alloy (HSLA) steel and AA6061-T6 welded by plasma arc and friction stir welding respectively. They explored that the microstructure of weld joint effects the mechanical properties quite significantly [13]. Numerous researches claimed that induced welding stresses adversely affect the product quality in operational life. Further, these stresses deteriorate mechanical properties and cause distortions in joints. In addition, different combinations of welding parameters, such as preheating, electrode diameter, weld sequence, groove angle, number of passes, heat input, job thickness etc. are investigated by experimentation to explore their individual and joint effect on welding distortions and joint properties [14,15]. In addition, it is claimed that welding distortions are unavoidable and its consequences cannot be ignored. Attempts are made to formulize a mathematical relationship to predict angular distortion in the steel structure. Further, welding distortion cause assembly problems, that requires rectification, thereby increasing manufacturing and assembly costs significantly [16]. Amir et al. attempted Taguchi method to optimize angular distortion of SMAW on low carbon steel joints. The effect of root gap, welding current and grove types was investigated. Welding current was found to be significant factor. However, information of electrode diameter used is missing [17]. It is assumed in TGRA, that all quality characteristics are independent and assigned equal weights. However, in real cases, this deviation may occur and to triumph over these issues, Hotelling and Pearson developed principal component analysis (PCA), which calculates prioritized weights for each quality responses. Kumar et al. applied PCA in TGRA to optimize mechanical properties of silica fly ash composites [18]. PCA has been vastly applied in fields of EDM [19], weaving [20], welding [21,22], etc.

The local industry is facing the problem of identification and control of input process parameters to obtain a weld quality joint with desired specifications. Currently, the welding parameter setting was determined by traditional procedures that encompass an experimental trial and error method: which is a time-consuming and error-based development method. This paper is framed at identifying, evaluating, and optimizing the influence of SMAW parameters on response variables for alloy steel SA 516 Grade 70 by employing Grey relational analysis (GRA) coupled with principal component analysis (PCA). Nine experimental runs were performed based on an L9 Taguchi orthogonal array to access best parameters combination for response variables namely tensile strength (TS), impact energy (IE), hardness, and angular distortion (AD). With reference to the available literature and best knowledge of the author, the optimization of the SMAW process with selected parameters for desired responses by employing GRA coupled with PCA has not been reported yet. Therefore, this paper constitutes a definite and worthwhile contribution to novelty in the related literature.

This research presents firstly the material and process parameters selection followed by the Taguchi experimental design. Then, the analysis of experimental results by Taguchi S/N ratios and GRA coupled with PCA was discussed. Validation of experimental results through confirmatory experiments is carried out in the last section.

## 2. Materials and Methods

### 2.1. Work Piece Material

In this study, samples of 16 mm thick carbon steel plates of SA 516 Grade 70 were used in welding experiments with dimensions 220 mm length and 110 mm width. SA 516 grade 70 is Carbon-Manganese steel most extensively used as major structural component in the fabrication of pressure vessels, boilers and petroleum tanks due to its sound weld ability and adequate mechanical properties at high temperatures [23]. Table 1 depicts chemical composition of SA 516 grade 70 [24].

### 2.2. Parameters and Response Variables

SMAW process involves large number of parameters that affect joint performance. In this study, four process parameters were selected after an extensive literature review and trial experimentation.

#### 2.2.1. Tensile Strength

**Figure 1:**
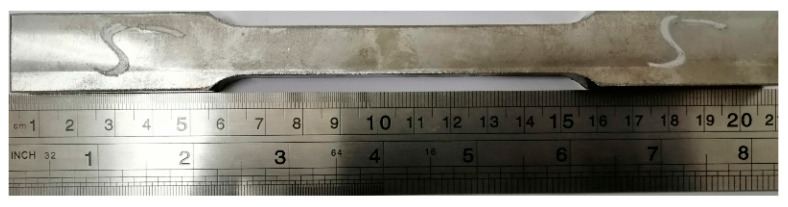
Tensile Test Specimen.

Specimens for tensile strength test as per A 370 standard were prepared. Tests were performed at room temperature on hydraulic Universal testing machine 50 ton capacity. The tensile test specimen is shown in Figure 1.

#### 2.2.2. Impact Energy

**Figure 2:**
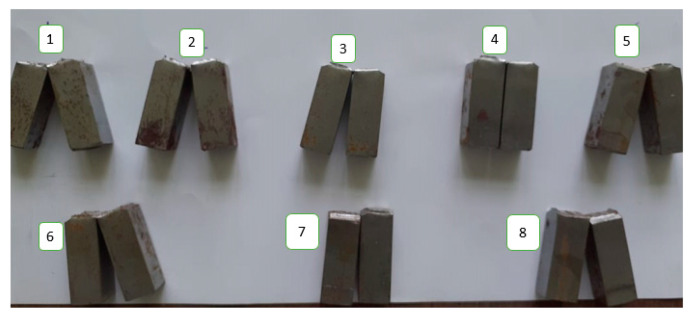
Charpy Impact Test Samples for: (1) Experiment 1; (2) Experiment 2; (3) Experiment 3; (4) Experiment 4; (5) Experiment 5; (6) Experiment 6; (7) Experiment 7; (8) Experiment 8.

To measure impact energy, the Charpy test was performed at an ambient temperature of 28–32 °C. Notch position was determined by macro-etching of samples in 2% Nital solution. The dimension of specimen was 55 mm × 10 mm × 10 mm. The Charpy impact test specimens for different experimental runs as expressed in Table 2 are shown in Figure 2.

#### 2.2.3. Hardness

Hardness was measured using portable Brinell hardness tester (EQUOTIP^®^, Schwerzenbach, Switzerland) as per ASTM A 370 standard [25]. The surface of work piece was polished and etched for ease in distinguish among weld zones. 

#### 2.2.4. Angular Distortion (AD)

**Figure 3:**
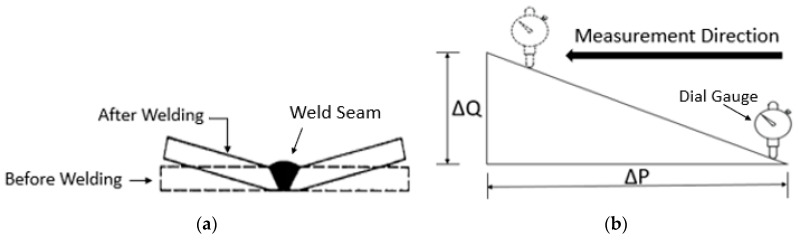
(**a**) Angular distortion; (**b**) Angular distortion measurement.

Angular distortion is the upward buckling of workpieces during welding due to the non-uniform rapid heating and cooling cycles during welding process induces residual stress in welded part. These residual stresses induce numerous types of distortions, adversely affects the performance of steel structures. Numerous problems such as dimensional inaccuracy, bending, decreased joint strength, buckling, and misalignments etc. Numerous techniques have been developed to overwhelm the adverse effects, however, techniques require tedious efforts and resource consumption [26]. AD of weld joint depends upon preheating, root gap, bead geometry groove angle, number of passes, plate thickness, heat input etc. [14]. AD decreases significantly by preheating the specimen, that lowers the joint residual stresses [15]. A dial gauge was used to measure angular distortion at different points of specimen [16]. Figure 3a,b depicts the angular distortion and its measurement respectively.

Using Figure 3b, the trigonometric relationship employed for determination of angular distortion in degrees, is expressed in Equation (1) [27].

(1) $$\theta =\tan^{-1}\frac{△Q}{△P}\;$$

## 3. Experimental Design

**Figure 4:**
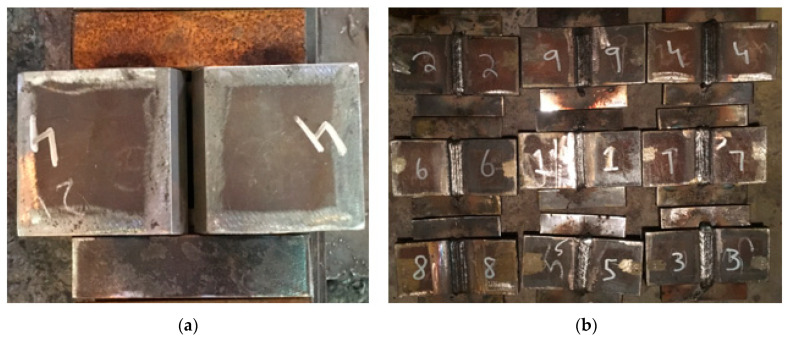
(**a**) Tacked Sample; (**b**) Welded Samples.

Taguchi orthogonal array become a valuable method for designing experiments to analyze quality characteristics and useful tool for obtaining highly reliable results, especially when the objective is the reduction of material cost and time [18,28]. Butt weld joints with single-V, square joint, double-V groove types are frequently adopted when the goal is a smooth surface. V type groove geometry is selected as it provides the best results for mechanical properties [29]. Tacked and welded samples are shown in Figure 4a,b.

The operating ranges of welding parameters chosen based on screening experiments and from American Welding Society (WPS) handbook and equally divided in three levels. Selected parameters and levels are depicted in Table 3.

The selected parameters are briefly defined in following sections

### 3.1. Groove Angle

Groove angle is a channel between two joining members that provides space for deposition of weld metal. It is the included angle between work pieces to be joined. Proper selection of groove angle significantly improves joint penetration, joint strength, and minimizes welding distortions. 

### 3.2. Preheating

Preheating is the process of heating work pieces to a predetermined temperature before commencement of welding operation. It is a form of heat treatment that plays a significant role governing joint properties. It is performed to retard the drastic cooling of HAZ and WM thereby greatly improves joint ductility and reduces the weld hardness. The profound effects of preheating are increase in grain size and depth of HAZ [10]. It also allows for diffusing absorbed hydrogen from WM and thereby helps in reducing the susceptibility of hydrogen induce cracking [30]. The dominant advantages of preheating are lowering residual stresses, moisture removal from joint, uniform expansion and contraction, and the improvement of fusion properties. In contrast, excessive preheating should be avoided as it induces thermal distortions.

### 3.3. Electrode Diameter

Electrode diameter significantly affects penetration depth and weld bead shape. At a specified current level, a smaller diameter electrode has a higher current density that results in high deposition. In contrast, electrodes of a larger diameter carry more amperage than a smaller one, and thus a larger diameter electrode deposits metals at higher rates.

### 3.4. Root Gap

Root gap is one of the initial geometrical features in welding of large steel structures that offers access to welding electrode and improves weld penetration to joining members. In order to obtain sound welding quality, the effect of root gap is necessity to be taken into account [31]. One of the emerging techniques for increasing productivity is narrow gap welding that significantly reduces number of passes. Root gap significantly effects welding distortion [31].

In this study, single V-Groove of three different angles was prepared by machining on the joining side of plates. Before welding, surfaces were grinded and cleaned to remove dirt and oxide scales. To provide same obstruction against angular distortion, ST-37 (low carbon steel) plates of dimensions 150 mm × 40 mm × 6 mm were tacked as fixture on both sides of plates. A butt joint was applied for welding in Flat (1 G) position by following the welding standards as per ASME IX [32]. The joint strength and economy was achieved by depositing root pass was using gas tungsten arc welding (GTAW), whereas hard, filling, and capping passes were performed by shielded metal arc welding (SMAW) [33].

The temper-bead-welding (TBW) technique is adopted for weld metal deposition as it significantly reduces residual stresses, hardness, deterioration of toughness properties. The travelling time for bead deposition in each layer was recorded. During experimentation, inter-pass temperature, polarity, electrode type, and welding speed were kept at 150 °C, direct current with positive polarity (DCEP), low hydrogen electrode E-7018, and 14–16 cm/min, respectively. To minimize spatter and undercut, the welding current and arc length were selected in accordance with requirements of electrode diameters for filling and capping passes.

Experimentation focused on changing in mechanical properties fusion zone and angular distortion of fabricated sample. Tensile, impact, and hardness tests were performed as per ASTM standard A 370 [34]. The test samples were sectioned across the welding direction in such a way that weld metal (WM) was in center of test coupons so that fractured encountered only in weld zone [35,36]. As three levels were set for each welding parameters, so the minimum of nine experimental runs were scheduled based on Taguchi orthogonal array L9 design, in order conserve resources and cost of experimentation. Table 2 depicts the Taguchi orthogonal array L9 design based on coded matrix, un-coded matrix, and experimental data.

## 4. Optimization Methodology

In this research work, Taguchi orthogonal arrays was employed for obtaining design matrix involving limited number of experiments that covers whole parametric space. Experiments are performed according to Taguchi orthogonal array design. The Taguchi method is popular commonly applied for optimizing of engineering problems; however, it is mono-optimization process [37,38,39], whereas several processes involve multiple response optimizations. Hence, the Taguchi method can’t tackle the optimization of multiple responses efficiently [40].

The larger, the better S/N ratio as computed from Equation (2):

(2) $$S/N\;ratio=\left(-10\right)\times \log_{10}\left(\frac{1}{x}\right)\overset{x}{\underset{i=1}{\sum }}\frac{1}{y_{ij}^{2}}$$

The smaller, the better S/N ratio as computed by Equation (3):

(3) $$S/N\;ratio=\left(-10\right)\times \log_{10}\left(\frac{1}{x}\right)\overset{x}{\underset{i=1}{\sum }}y_{ij}^{2}$$

where *x* is number of replications and $y_{ij}$ is measured observation.

**Figure 5:**
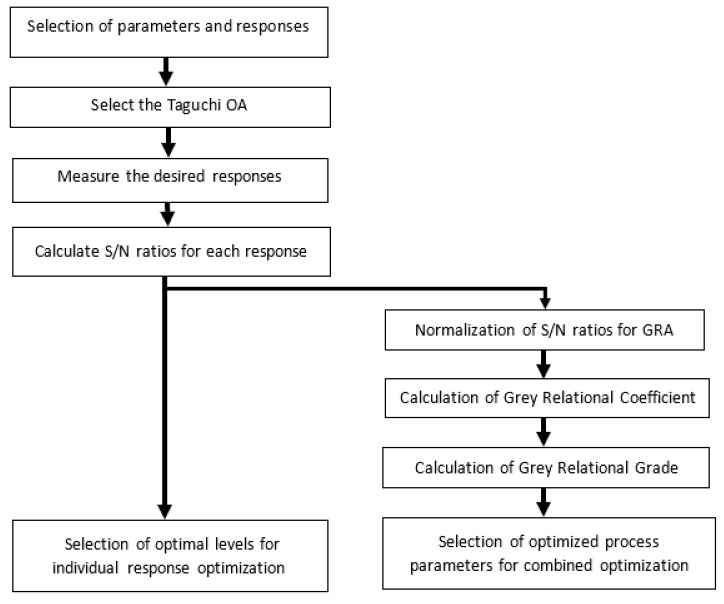
Concept behind PCA-GRA.

Welding process has multiple responses and welding quality sturdily depends upon optimizing all responses simultaneously. Therefore, researchers frequently employ GRA coupled with PCA for optimization of multiple responses simultaneously. These techniques are entirely different to traditional single response optimization. These are effective statistical methods and offer quite successful results in obtaining a combination of parameters for multiple response optimizations [41]. Figure 5 depicts the concept of PCA-GRA.

In 1982, Deng proposed GRA method that is principally employed for analyzing the effect of process parameters on multiple responses where information is deficient, and system is ambiguous. GRA initiates with Grey relational generation [42], which involves the linear normalization of experimentally collected data (reference sequence) in a range between 0 and 1 (comparable sequence).

Depending upon the objective of this paper, the maximization of tensile strength and impact energy is of interest. Therefore, larger-the-better criterion is selected for these quality characteristics and normalized results can be expressed as Equation (4)

(4) $$y_{j}^{*}\left(q\right)=\frac{y_{j}\left(q\right)-min\;y_{j}\left(q\right)}{max\;y_{j}\;\left(q\right)-min\;y_{j}\left(q\right)}$$

Further, hardness and angular distortion need to be minimized, thus the smaller-the-better is used, as expressed in Equation (5)

(5) $$y_{j}^{*}\left(q\right)\;\frac{max\;y_{j\;}\left(q\right)-y_{j}\left(q\right)}{max\;y_{j}\;\left(q\right)-min\;y_{j}\left(q\right)}$$

where $y_{j}^{*}$(*p*) are the generated grey relational values, while *max* $y_{i}\left(q\right)$ and *min* $y_{i}\left(q\right)$ are the largest and smallest values of $y_{j}$(*q*) for qth observation, respectively. *q* = 4 is the number of response variables. The nine observations of the experiments are comparability sequence $y_{i}\left(q\right),\;j=1,2,\ldots ,9,$. The best normalized results should be equal to 1, therefore; for achieving better performance, larger value of normalized results is expected.

Data normalization is followed by calculation of grey relational coefficients (GRC) that displays the relationship between desirable and real experimental normalized results. Expression of GRC $\xi_{j}\left(q\right)\;$is determined, as follows in Equation (6)

(6) $$\xi \left(y_{j}^{*}\left(q\right),y_{0}^{*}\left(q\right)\right)=\frac{\Delta_{min}\left(q\right)+\zeta \;\Delta_{max\;}\left(q\right)}{\Delta_{0j}\;\left(q\right)+\Delta_{max}\;\left(q\right)}$$

where $\Delta_{0i}\left(q\right)=\left|y_{0}^{*}\left(q\right)-y\left(q\right)\right|$ is deviation sequence, defined as absolute of difference between reference sequence $y_{0}^{*}\;\left(q\right)$ and comparability sequence $y_{j}^{*}\;\left(q\right).$ The identification or distinguishing coefficient (𝜁), takes value as 𝜁 𝜖 [0, 1], which is generally and in this paper were set as 0.5 [43]. Grey relational grade (GRG) provides information about correlation strength between the experimental runs, which is computed by weighted mean of respective GRC’s for all experimental. GRG value lies between 0 and 1, γ 𝜖 [0, 1]. Usually, an experimental run with larger GRG is considered the ideal case, which indicates the strength of correlation between corresponding experiments and the ideally normalized value. When equal weights are opted for all quality responses, Equation (7) is used for GRG calculation.

(7) $$\gamma_{j}\left(y_{0}^{*},y_{j}^{*}\right)=\frac{1}{n}\;\overset{n}{\underset{q=1}{\sum }}\xi \left(y_{j}^{*}\left(q\right),y_{0}^{*}\left(q\right)\right)$$

In some applied applications, weights of quality characteristics are different likewise weights obtained from PCA. In such cases, Equation (7) is modified as Equation (8) [44]:

(8) $$\gamma_{j}\left(y_{0}^{*},y_{j}^{*}\right)=\frac{1}{n}\;\overset{n}{\underset{q=1}{\sum }}w_{q}\;\xi \;(y_{j}^{*}\left(q\right),y_{0}^{*}\left(q\right))$$

where $\gamma_{j}\left(y_{0}^{*},y_{j}^{*}\right)$ is GRG for $j^{th}$ experimental run, n is number of quality response, $w_{q}$ is weight of $q^{th}$ quality response and $\overset{n}{\underset{q=1}{\sum }}w_{q}=1$.

### Principal Component Analysis (PCA)

PCA is a powerful multivariate statistical technique for multi-objective optimization [20] that reduces the complexity, correlation, vagueness, and dimensions of information by simplifying and combining numerous allied arrays into few uncorrelated arrays and principal component. PCA employs linear permutation for conserving unique information to maximum extent [45]. Thus, it converts multi-response optimization to single response optimization without compromising original information [46]. It begins by setting a structure of linear combinations arrays of multi-responses. The GRC’s computed for response variables is employed to form a matrix, presented as Equation (9)

(9) $$y=\left[\begin{array}{cccc} y_{1}\left(1\right) & y_{1}\left(2\right) & \cdots & y_{1}\left(k\right)\\ y_{2}\left(1\right) & y_{2}\left(2\right) & \cdots & y_{2}\left(k\right)\\ \ldots & \ldots & \ldots & \ldots \\ \ldots & \ldots & \ldots & \ldots \\ y_{j\;}\left(1\right) & y_{j}\left(2\right) & \ldots & y_{j}\left(k\right) \end{array}\right]$$

where $y_{p}\left(q\right)$ is GRC of each quality responses, *p* = 1, 2,…*j*, experiments and *q* = 1,2, … *k*, quality responses. In this research, *j* = 9 and *k* = 4. Thereafter, the coefficient correlation matrix can be generated by the following expression:

(10) $$R_{jl=}\left(\frac{Cov\;\left(y_{p}\left(q\right),y_{p}\left(l\right)\right)}{\sigma_{yp}\left(q\right)\;*\sigma_{yp}\left(l\right)}\right)\;q=1,2,\ldots k;\;l=1,2,\ldots ,k$$

where $Cov(y_{p}\left(q\right),y_{p}\left(l\right)$ is the covariance of sequences $y_{p}\left(q\right)$ and $y_{p}\left(l\right)$.$\sigma_{yp}\left(q\right)$ is standard deviation of sequence $y_{p}\left(q\right)$ and $\sigma_{yp}\left(l\right)$ is standard deviation of sequence $y_{p}\left(l\right)$. The eigen values and eigen vectors are computed from $R_{jl}$ array as per Equation (11)

(11) $$(R-\lambda_{k}I_{j})\;V_{pk}=0$$

Thereafter, eigenvalues $\left(\lambda_{k}\right)$ and eigenvectors ($V_{pk})$ of square matrix R are used to determine the uncorrelated principal components (PC’s) by using Equation (12)

(12) $$Z_{jk}=\overset{n}{\underset{i=1}{\sum }}Y_{j}\left(p\right)\times V_{pk}$$

where $Z_{jk}$ corresponds to kth principal component. Eigenvalues and principal components are arranged in descending order with respect to explained variance, therefore, first eigenvalue associated with first PC accounts for largest variance contribution. Eigenvalues corresponding to eigenvectors are presented in Table 4.

## 5. Results and Discussion

### 5.1. Probability Plots

**Figure 6:**
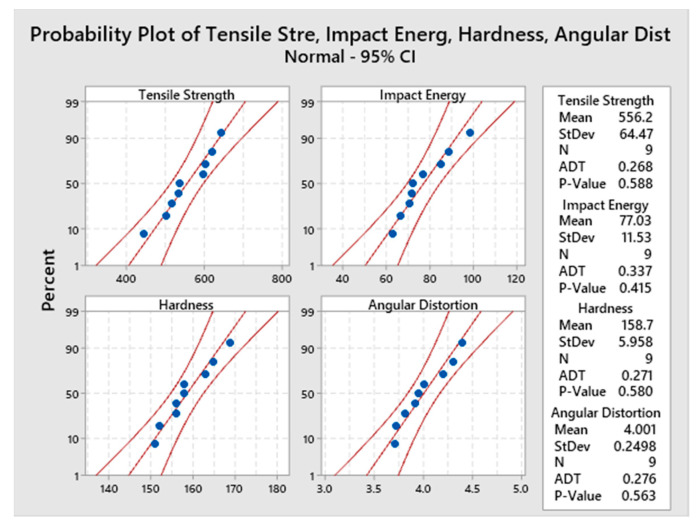
Normal probability plots of responses.

Probability plots measures the distribution of experimental data, as tabulated in Table 2. The Anderson Darling (ADT) test, a powerful statistical tool generally employed for outlier detection from normality, is employed for validation of normality assumption [47]. Figure 6 shows that the experimental data for all responses falls near the fitted line, and the Anderson Darling (ADT) statics values are relatively low and *p*-value of the test are greater than 0.05 so it is assumed that the data follows normal distribution. Therefore, further analysis and optimization can be performed on the data.

### 5.2. ANOVA and Main Effect Plots of Means for Individual Responses

The analysis of variance (ANOVA) is performed at 95% confidence interval to study the main effect of input parameters on individual response. The ANOVA results for tensile strength, impact energy, hardness and angular distortion are expressed in Table 5. *p*-value less than 0.05 shows significance of parameter.

**Figure 7:**
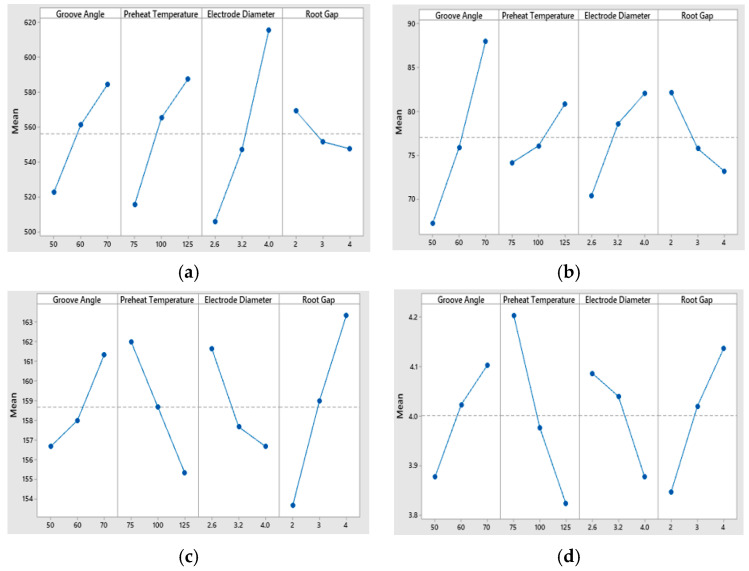
Main effect plot of means for (**a**) tensile strength; (**b**) impact energy; (**c**) hardness; (**d**) angular distortion.

In the case of tensile strength, the most influencing parameter is electrode diameter (ED) with % contribution of 55.18%, followed by preheat temperature (PHT), i.e., 23.38%, groove angle (GA), i.e., 16.55%, while root gap (RG) is found to be insignificant, having the least % contribution of 2.11% and *p*-value greater than 0.05. Figure 7a shows the main effect plot of means for tensile strength. It shows that tensile strength increases with increase in groove angle, preheat temperature, and electrode diameter from low level to high level, i.e., 50–70°, 75–125 °C, and 2.6–4.0 mm, however decreases with increase in root gap from low level to high level, i.e., 2–4 mm. The results are in line with the studies [48,49,50]. In all trails of tensile tests, samples were fractured from base metal, this indicates high joint strength that may be due to presence of acicular ferrite in WM which imparts high strength [51]. An overall increase in WM tensile strength was noticed. Main effects plots of tensile strength are shown in Figure 7a.

For impact toughness GA is significant with % contribution of 61.03% followed by ED (18.32%), RG (11.30%) and PHT (6.31%). From Figure 7b, it is evident that impact energy increases dramatically with increase in groove angle from low level to high level. Similarly, it increases steadily with an increase in PHT and ED from low level to high level. In contrast, impact energy drops linearly with increase in root gap from low level to high level. These results are in line with findings of literature [52,53]. Main effects plots of impact energy are shown in Figure 7b.

For hardness, the order of significance is RG (49.35%), PHT (23.47%), ED (12.37%), and GA (12%). It is evident from Figure 7c that hardness decreases sharply with an increase in PHT and ED, Further, hardness increases with an increase in GA and RG. Hardness noted at WM zone was nearly 7% percent higher than base metal and fluctuation among measured values also witnessed. This high hardness and scattering could be attributed to smaller grain size, metallurgical changes, difference in carbon content and rapid cooling rates as reported [54]. Highest hardness in observed in WM, followed by HAZ and BM. The higher value of hardness in WM zone can be attributed to presence Widmanstatten ferrite. The hardness of samples varied from 161–195 HB. In contrast with WM hardness, lower HAZ hardness is may be due to preheating. The results are in line with findings of the literature [34]. Main effects plots of hardness are shown in Figure 7c.

Finally, for angular distortion the most contributing factor is PHT (43.38%), followed by RG (25.26%), GA (14.64%), and ED (13.86%). From Figure 7d, it is evident that angular distortion increases sharply with an increase in GA and RG, Further, AD decreases with increase in PHT and ED. The results are in line with work of [17,55]. Main effects plots of angular distortion are shown in Figure 7d. Table 4 depicts the ANOVA results for quality responses.

The effect of selected welding parameters on response variables are expressed in Section 5.2.1, Section 5.2.2, Section 5.2.3 and Section 5.2.4

#### 5.2.1. Groove Angle

A sharp increase in impact strength with increase in included angle, this is due to increase in volume of deposited weld metal. In this research hardness value raised with the increment in groove angle, this is due to increase in volume requirements of filler metal to fill groove [56]. Secondly, increase in surface area of molten metal weld pool causes rise in cooling rate that reduces grain growth time and rapid development of fine grains that increase hardness [57]. Larger included weld groove angles have greater volume. Hence, larger filler metal deposition is required to fill them. This greater deposition involves more expansion and contraction cycles resulting in generation of greater induced residual stresses. The magnitude of angular distortion become very large and effects structural integrity.

#### 5.2.2. Preheating

Preheating of work piece limits the cooling rate that directly influence tensile strength, impact toughness and harness of weld joint. As a result, weld metal got more time to fuse evenly with base metal thereby improves joint integrity. Increase in preheat temperature changes the mode of fracture in weld joints from brittle to ductile. The increase in preheat temperature promotes formation of pearlite and ferrite that Low preheat temperature shortens the cooling time which leads to intensive hardening, cold cracking and joint embrittlement [58]. The preheating significantly mitigates residual stresses and bend up angular distortion induced by the cumulative plastic strain. The two main effects of preheating are: firstly, the minimization of angular distortion through reduction in temperature gradient in workpiece that allows for the homogenous contraction of material on cooling. Secondly, it slows down cooling rates that limits volumetric fraction of martensite and promotes bainite fraction. Since the bainite volume is greater than martensite volume [59], this lowers the induced tensile stresses, leads to small distortion amplitude, and hence promotes high quality welds. The results are in line with authors [60].

#### 5.2.3. Electrode Diameter

It is observed that smaller diameter electrodes increase heat input that leads to coarsening of grains results brittleness of joint. The higher heat input also produces significant variation in microstructure of WM and HAZ, thus coarsening of grains take place that lead to deterioration of tensile and impact strength and increase in hardness [61]. Therefore, it is crucial to restrict the heat input of welding to limited range. With the increase in electrode diameter from 2.6 mm to 4 mm, the angular distortion decreases significantly, and this is due to reduction in number of welding passes and heat input requirement for complete penetration. Further, for same heat input and groove angle, increase in number of passes due to smaller electrode diameter leads to significantly higher angular distortions. Further, TWI considers magnitude of angular distortion approximately proportion to number of welding passes [62].

#### 5.2.4. Root Gap

An increase in root gap causes higher heat input. This increases tendency of grain growth to have adverse effects on the tensile strength of WM [63]. The higher root gap results in higher heat input that cause enlargement of HAZ which adversely effects impact energy of weld specimen [64]. Increase in root gap greatly increases depth of weld penetration that increases hardness of weld joint [63]. It is observed that increase in root gap involve as many weld passes, the heating and cooling cycles during each weld pass induces higher bend stress and different final stresses. This extends the distribution of weld residual stresses over wide range. In specimens with a 4 mm root gap, lifting of the specimen from both sides is greater than specimens with 2 mm and 3 mm root gaps [64]. Larger root gaps significantly increase lateral shrinkages, thus resulting in axial displacements and buckling of jobs [65]. Contrary, smaller root gaps minimize weld deposition volume, which reduces number of welding passes leading to less heat input and results in less shrinkages and distortions [66]. Further, samples fabricated with root gap of 3 mm and 4 mm are encountered with sagging defect which indicates that welding heat input is slightly higher than required. Full penetration is obtained at all three root gaps. In a root gap of 2 mm, a good surface is obtained on capping and root surfaces.

### 5.3. Single Objective Optimization

**Figure 8:**
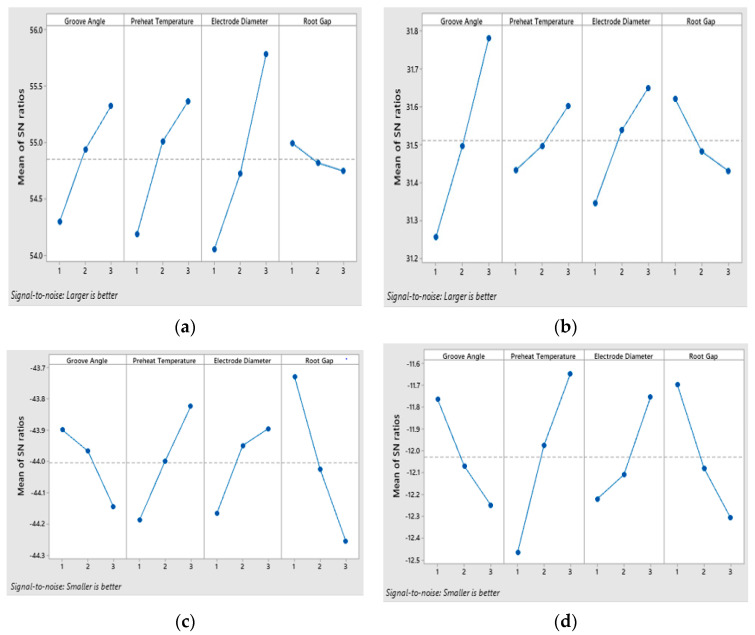
Main effect plot of SN ratios for (**a**) tensile strength; (**b**) impact energy; (**c**) hardness; (**d**) angular distortion.

Individual responses are optimized based on signal to noise (S/N) ratios. In present study we have conflicting objective functions of individual responses, i.e., maximization for tensile strength and impact energy and minimization for hardness and angular distortion. Therefore, the larger-the- better-quality characteristic is applied to tensile strength and impact energy using Equation (1), while the smaller-the-better quality characteristic is applied to hardness and angular distortion using Equation (2). Optimal levels are obtained by computing the average values of S/N ratios for each response at each level as shown in Figure 8. Higher values of S/N ratios show good quality characteristics. Table 6 shows that higher S/N ratio of 56.16 and -43.58 are obtained for tensile strength and hardness at experimental run 5 having groove angle and preheat temperature at level 2, electrode diameter at level 3, and root gap at level 1. For impact energy, the higher S/N ratio observed is 39.87 at experiment 9 having a groove angle and preheat temperature at level 2, electrode diameter at level 2, and root gap at level 1. For angular distortion the higher S/N ratio computed is −11.39 at experimental run 3 having groove angle at level 1 while preheat temperature, electrode diameter, and root gap at level 3 respectively. Figure 8a,b shows the optimal levels for tensile strength and hardness, which are groove angle at level 3, preheat temperature at level 3, electrode diameter at level 3, and root gap at level 1. Figure 8c,d shows optimal levels for impact energy and angular distortion which are groove angle and root gap at level 1, preheat temperature and electrode diameter at level 3.

It is evident from Table 5 that huge inconsistency lies among the optimal setting for all responses. Therefore, the need of multi objective optimization arises.

### 5.4. Multi Response Optimization based on GRA and PCA

In GRA, all response variables are assigned equal weights, which may cause uncertainty in decision making. Therefore, PCA is employed to determine relative weights of quality responses [67]. In this research work, it is attempted to compare multi objective optimization performed by GRA and PCA and to validate results by confirmatory experiments.

The steps are discussed in detail in the optimization methodology section. First, the S/N ratios depicted in Table 2 are normalized using Equations (4) and (5). The Grey relational coefficient of individual responses is computed using Equation (6). Un-weighted grey relational grade is calculated using Equation (7) and presented in Table 7.

For PCA, the Eigen values and Eigen vectors are calculated by Equation (11) and PC components from Equation (12), presented in Table 4. The relative weights of quality responses are obtained by squaring the Eigen vectors of first PC. Using the calculated weights from PCA and GRCs tabulated in Table 6, W-GRGs are computed for nine experiments using Equation (8). The W-GRGs are ranked and presented in Table 6.

In Table 6, highest GRG and W-GRG value is obtained at sample no. 8. Further, in Table 8, means of GRG and W-GRG depicts identical optimal conditions. From Table 4, it is evident that first PC accounts as high as 61.4% variance contribution for four quality characteristics. Table 9 presents the squares of eigenvectors of the first PC that are chosen as weights of quality responses that are found to be equal to 0.2777, 0.1672, 0.2714, and 0.284 for tensile strength, impact energy, hardness, and angular distortion, respectively. Table 10 explains comparison of W-GRA (GRG: 0.7645) with initial conditions and found W-GRG value improved by 41.12%. Thus, the desirable multi objective optimization can be achieved with respect to single GRG. Thus, based on GRG and W-GRGs, the optimum set of input parameters levels for quality responses is A_3_B_1_C_3_D_3_, namely groove angle 70° (level 3), preheat temperature 75 °C (level 1), electrode diameter 4 mm (level 3), and root gap 4 mm (level 3).

## 6. Confirmation Experiment

Confirmation experiment was conducted on optimal levels of welding parameters identified by GRA and W-GRA to evaluate and verify the improvement in quality response of SMA weld joint on SA 516 grade 70. The predicted value of GRG, namely $\gamma_{predicted}$, at optimal levels of parameters is calculated by following the expression as shown in Equation (13) [18]:

(13) $$\gamma_{predicted}=\gamma_{m}+\overset{n}{\underset{i=1}{\sum }}\left(\gamma_{i}-\gamma_{m}\right)$$

where $\gamma_{m}$ is mean GRG, $\gamma_{i}$ is mean GRG at optimal level of $i^{th}$ welding parameter, and n are the welding parameters that significantly affect quality responses. It is obvious from confirmatory results that highest values of tensile strength and impact strength, whereas the lowest values of hardness and angular distortion are achieved.

The predicted W-GRG from Equation (13) is 0.7543. Thereafter, confirmatory experiments are performed at the optimal settings predicted by GRA and W-GRA to validate the results of both techniques and the experimental value obtained is 0.7645. Since, the GRG value improved by 0.2228 (41.12%), it is obvious that there is a good agreement between predicted and experimental values. Based upon GRA and W-GRG results, a significant improvement in tensile strength by 23.80%, improvement in impact energy by 64.38%, reduction in hardness by 3.01%, and reduction in angular distortion by 7.14% have been found. Hence, GRA and PCA based GRA are found to be useful approaches for multi-objective optimization problems. The confirmation results of tensile strength, impact energy, hardness and angular distortion are depicted in Table 10.

Results of confirmatory experiments are quite satisfactory and pronounce improvements in quality responses was observed. Process settings of GRA and W-GRA are mostly similar.

## 7. Microstructure

**Figure 9:**
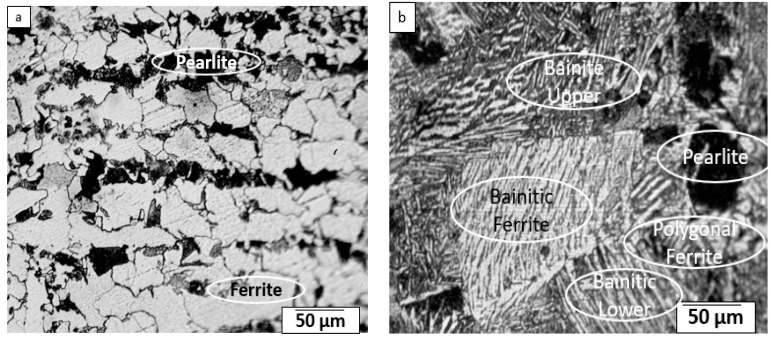

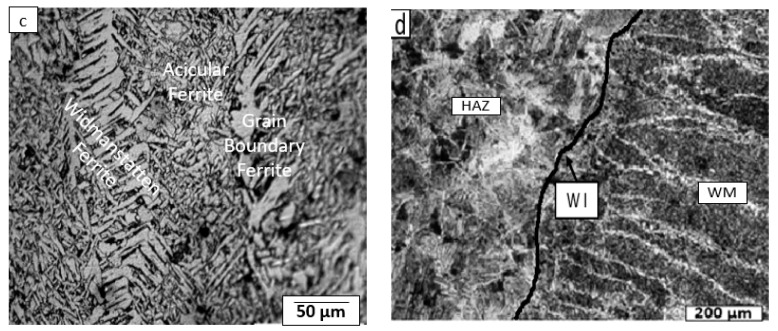
SA 516 Grade 70 Microstructure of (**a**) BM; (**b**) WM; (**c**) HAZ; (**d**) WM/HAZ interface.

Microstructure examination was performed at base metal (BM), weld metal (WM), heat effected zone (HAZ) and WM/HAZ interface using optical microscope. Standard procedure for preparation of metallographic samples is followed, that includes mechanical grinding by silicon carbide emery paper of grit sizes (80, 160), polishing, and etching with 4% Nital solution. Required size of BM, WM, HAZ, and WM/HAZ interface was sectioned and mounted. It is observed that parent metal consists of ferrite content 70% and pearlite content 30%. WM or FZ microstructure reveals presence of acicular ferrite (AF), grain boundary ferrite (GF) and some proportion of Widmanstätten ferrite (WF). WF is formed by nucleation of ferrite side plate at boundaries of austenite/ferrite into austenite. The presence of AF in WM contributes to high toughness and strength [34,68]. Further, the tenacity of weld joint is attributed to the presence of AF in WM, that also ensures gain in mechanical properties [34]. In HAZ, formation of bainite, pearlite and fine ferrite was found. Pearlite surrounded by ferrite where carbon percentage was 0.45%. Mechanical properties as well as microstructure primarily depend upon heat input, chemical composition of base metal, cooling rate, initial grain size, phases, and electrode composition [69]. Present study results are similar and agree to reported by Pritesh [70]. The microstructure of BM, WM, HAZ, and WM/HAZ are presented in Figure 9.

## 8. Conclusions

This investigation attempts to solve multiple quality-response parametric optimization of SMAW. Initially, nine experiments were designed and conducted as per Taguchi L9 OA, followed by application of GRA-integrated-PCA approach for extracting optimal solution of complicated multi objective optimization problem. PCA was utilized to extract weightages for quality responses that influence GRGs. Eventually, confirmatory experiments were conducted to cross check the optimal setting. Obtained critic metrics from present research are

1. To achieve multiple objective optimization of SMAW process for pressure vessel steel SA 516 grade 70, the optimal combination of parameters is GA_3_PHT_1_ED_3_RG_3_.
2. The percentage contributions of each quality response for principal component in decreasing order are angular distortion (28.40%), tensile strength (27.79%), hardness (27.14%), and impact energy (16.72%) respectively.
3. The analysis of the average of GRG revealed that groove angle has the maximum influence, followed by electrode diameter, root gap, and preheat temperature, respectively.
4. The analysis of the average of W-GRG revealed that groove angle has maximum influence, followed by root gap, preheat temperature, and electrode diameter, respectively.
5. GRA and W-GRG identified identical optimal combination of input parameters as: groove angle 70°; preheat temperature 75 °C; electrode diameter 4 mm; and root gap 4 mm.
6. Significant improvement in GRG from initial condition to optimal setting is found as 0.2898 as is achieved by GRA approach.
7. Finally, a confirmatory experiment on GRG/W-GRA based optimal settings showed an improvement of 23.80% in tensile strength, 64.38% in impact energy, 3.01% in hardness, and 7.14% in angular distortion.

The results of GRA and W-GRA methods are compared and found same optimal settings for both techniques. Research work findings can be used as guidelines and standards for SMAW of pressure vessels in practical applications. Moreover, future work of research lies in exploring effects of quantitative and qualitative inputs on other outputs, such as bead height, bead reinforcement, penetration, residual stresses etc. Finally, W-GRA is found to be an easy, simple, effective, and efficient algorithm for stake holders of the welding world. Future work on this may concentrate on finite element analysis, with a focus on other parameters, tests, and statistical techniques.

## Figures and Tables

**Table 1:** Chemical composition of ASME SA 516 Grade 70.

Element	C	Al	V	Cr	P	Mn	Si	Sn	N	As	S	Cu
% by weight	0.22	0.039	0.002	0.03	0.018	0.99	0.18	0.002	0.005	0.002	0.008	0.02

**Table 2:** Design Matrix and Experimental Results.

Exp. No.	Coded Matrix				Un-Coded Matrix				Experimental Result			
	A	B	C	D	GA	PHT	ED	RG	TS (MPa)	IE (J)	H (HB)	AD (θ)
1	1	1	1	1	50	75	2.6	2	445	62.88	158	4.01
2	1	2	2	2	50	100	3.2	3	518	66.59	156	3.91
3	1	3	3	3	50	125	4.0	4	605	72.28	156	3.71
4	2	1	2	3	60	75	3.2	4	503	70.77	165	4.4
5	2	2	3	1	60	100	4.0	2	643	85.10	151	3.72
6	2	3	1	2	60	125	2.6	3	538	71.80	158	3.95
7	3	1	3	2	70	75	4.0	3	599	88.84	163	4.2
8	3	2	1	3	70	100	2.6	4	535	76.56	169	4.3
9	3	3	2	1	70	125	3.2	2	620	98.48	152	3.81

**Table 3:** Welding parameters and levels.

Parameters	Symbol	Units	Level 1	Level 2	Level 3
Groove Angle	A	Θ	50	60	70
Pre-Heat Temperature	B	°C	75	100	125
Electrode Diameter	C	mm	2.6	3.2	4
Root Gap	D	mm	2	3	4

**Table 4:** Principal component analysis.

Component	PC 1	PC 2	PC 3	PC 4
Eigen Value	2.4549	1.1014	0.2804	0.1634
Variation (%)	0.614	0.275	0.070	0.041
Cumulative (%)	0.614	0.889	0.959	1.000
				
Eigen Vector	−0.527	0.390	−0.705	−0.272
	−0.409	0.662	0.592	0.207
	0.521	0.461	−0.371	0.615
	0.533	0.444	0.121	−0.710

**Table 5:** ANOVA for individual responses.

	Source	DoF	Adj SS	Adj MS	F-Value	*p*-Value	% Contribution
Tensile strength	Groove angle	1	5504.6	5504.6	23.98	0.008	16.55
	Preheat temperature	1	7776	7776	33.88	0.004	23.38
	Electrode diameter	1	18351.4	18351.4	79.97	0.001	55.18
	Root gap	1	704.2	704.2	3.07	0.155	2.11
	Error	4	918	229.5			2.76
	Total	8	33253.6				100
Impact energy	Groove angle	1	648.99	648.99	80.68	0.001	61.03
	Preheat temperature	1	67.13	67.13	8.35	0.045	6.31
	Electrode diameter	1	194.79	194.79	24.21	0.008	18.32
	Root gap	1	120.154	120.154	14.94	0.018	11.3
	Error	4	32.18				3.02
	Total	8	1063.24				100
Hardness	Groove angle	1	34.105	34.105	17.24	0.014	12
	Preheat temperature	1	66.667	66.667	33.7	0.004	23.47
	Electrode diameter	1	35.149	35.149	17.77	0.014	12.37
	Root gap	1	140.167	140.167	70.86	0.001	49.35
	Error	4	7.913	1.978			2.78
	Total	8	284				100
Angular distortion	Groove angle	1	0.0731	0.0731	20.57	0.011	14.64
	Preheat temperature	1	0.2166	0.2166	60.95	0.001	43.38
	Electrode diameter	1	0.06923	0.06923	19.48	0.012	13.86
	Root gap	1	0.12615	0.12615	35.5	0.004	25.26
	Error	4	0.01421	0.00355			2.84
	Total	8	0.49929				100

**Table 6:** Experimental design and results using L9 OA.

Exp. No.	S/N Ratios of Responses			
	Tensile Strength	Impact Energy	Hardness	Angular Distortion
1	52.97	35.97	−43.97	−12.06
2	54.29	36.47	−43.86	−11.84
3	55.64	37.18	−43.86	−11.39 *
4	54.03	37.00	−44.35	−12.87
5	56.16 *	38.60	−43.58 *	−11.41
6	54.62	37.12	−43.97	−11.93
7	55.55	38.97	−44.24	−12.46
8	54.57	37.68	−44.56	−12.67
9	55.85	39.87 *	−43.64	−11.62
Optimum	A_2_B_2_C_3_D_1_	A_3_B_3_C_2_D_1_	A_2_B_2_C_3_D_1_	A_1_B_3_C_3_D_3_

* optimized setting for individual responses.

**Table 7:** Calculated Normalized, GRC and GRG for 9 experiments.

Exp No.	Normalization				Grey Relational Coefficient				GRG	Rank	W-GRG	Rank
	TS	IE	H	AD	TS	IE	H	AD				
1	0.00	0.00	0.40	0.46	0.33	0.33	0.46	0.48	0.400	9	0.408	9
2	0.41	0.13	0.29	0.31	0.46	0.36	0.41	0.42	0.414	8	0.419	8
3	0.83	0.31	0.29	0.00	0.75	0.42	0.41	0.33	0.479	6	0.485	6
4	0.33	0.26	0.79	1.00	0.43	0.40	0.70	1.00	0.633	4	0.661	3
5	1.00	0.67	0.00	0.02	1.00	0.61	0.33	0.34	0.569	5	0.565	5
6	0.52	0.30	0.40	0.37	0.51	0.42	0.46	0.44	0.455	7	0.459	7
7	0.81	0.77	0.68	0.73	0.72	0.69	0.61	0.65	0.665	2	0.664	2
8	0.50	0.44	1.00	0.87	0.50	0.47	1.00	0.79	0.689	1	0.712	1
9	0.90	1.00	0.06	0.16	0.83	1.00	0.35	0.37	0.638	3	0.598	4

**Table 8:** Response table for average GRG and W-GRG.

Parameters	Average of GRG					Average of W-GRG				
	Levels			Delta	Rank	Levels			Delta	Rank
	1	2	3			1	2	3		
GA	0.431	0.552	0.664 *	0.2334	1	0.437	0.561	0.658 *	0.220	1
PHT	0.566 *	0.557	0.524	0.0423	4	0.577 *	0.566	0.514	0.063	3
ED	0.515	0.562	0.571 *	0.0564	3	0.526	0.560	0.571 *	0.063	4
RG	0.535	0.511	0.601 *	0.0893	2	0.524	0.514	0.619 *	0.105	2

* optimized setting for individual responses.

**Table 9:** Variance contribution of response variables for first PC.

Response Variable	Contribution
Tensile Strength	0.2777
Impact Energy	0.1672
Hardness	0.2714
Angular Distortion	0.2840

**Table 10:** Comparison Summary of confirmatory experiments.

	Initial Condition A_2_B_2_C_3_D_2_	Confirmatory Experiment Results	Improvement from Initial Condition (%)
		GRG/W-GRA	GRG/W-GRA
Tensile Strength (MPa)	545.6	675.5	23.80
Impact Energy (J)	60.76	99.88	64.38
Hardness (HB)	166	161	3.01
Angular Distortion (θ)	4.2	3.9	7.14
Optimal Condition	-	A_3_B_1_C_3_D_3_	
Grey Relational Grade	0.5417	0.7645	
